# Deciphering the Germination Behavior of Sesame Cultivars: The Interplay of Hydrothermal Time Model Parameters and Seed Fatty Acid Profiles

**DOI:** 10.3390/plants14223422

**Published:** 2025-11-08

**Authors:** Hamidreza Balouchi, Vida Soltani Khankahdani, Ramin Piri, Ali Moradi, Majid Gholamhoseini, Seyedeh Zahra Heydari, Lina Q. Ahmed, Abraham J. Escobar-Gutiérrez, Beata Dedicova

**Affiliations:** 1 Department of Agronomy and Plant Breeding, Yasouj University, Yasouj 75918-74831, Iran; vidasoltani73@gmail.com (V.S.K.); amoradi@yu.ac.ir (A.M.); shdy.hy2000@gmail.com (S.Z.H.); 2 Department of Agronomy and Plant Breeding, University of Tehran, Tehran 31587-77871, Iran; raminpiri88@yahoo.com; 3 Department of Oilseed Research, Seed and Plant Improvement Institute, Agricultural Research, Education and Extension Organization (AREEO), Karaj 31359-33151, Iran; m.gholamhoseini@areeo.ac.ir; 4 UR4 P3F, INRAE, Unité de Recherche Pluridisciplinaire Prairies et Plantes Fourragères, Le Chêne-BP 6, F-86600 Lusignan, France; lina-qadir.ahmed@inrae.fr (L.Q.A.); abraham.escobar-gutierrez@inrae.fr (A.J.E.-G.); 5 Department of Plant Breeding, Swedish University of Agricultural Sciences (SLU), Alnarp, Sundsvägen 10, P.O. Box 190, SE 234 22 Lomma, Sweden

**Keywords:** basal water potential, hydrothermal time, seed oil, germination

## Abstract

Seed germination is a critical developmental stage susceptible to environmental factors, such as temperature and soil water potential, which directly influence germination percentage and rate. To gain deeper insight into these interactions, this study re-analyzed data from a previously published germination experiment involving six distinct sesame (*Sesamum indicum* L.) cultivars with varied fatty acid profiles. Germination was recorded across eight constant temperatures (10–45 °C) and seven water potential levels (0 to −1.2 MPa), using thermal time, hydrotime, and hydrothermal time models. No germination occurred at −1.0 or −1.2 MPa. The highest thermal time constant, θT(g), was observed at −0.8 MPa across all cultivars, and germination rates declined as water potential decreased. Hierarchical clustering based on principal component analysis grouped the cultivars into three distinct clusters. Cluster 1, containing two cultivars, exhibited the highest base temperatures (above 13 °C), which correlated with high arachidic and low linolenic acid content. The three cultivars in cluster 2 showed the highest hydrothermal time constants (θHT(g) = 760–2495 MPa·°C·h) and contrasting palmitic and stearic acid profiles. The single cultivar in cluster 3 had the lowest base water potential, the highest oleic acid content, and the highest percentage of linoleic acid. These findings underscore the significant role of seed fatty acid composition in stress tolerance, with ‘Darab1’ showing superior drought resistance and ‘Dashtestan2’ showing greater resilience to high temperatures. These modeling and multivariate analyses provide deeper insights into germination mechanisms and offer valuable guidance for selecting cultivars adapted to specific climatic conditions.

## 1. Introduction

Environmental conditions during maternal plant development can alter the oil content and fatty acid composition in the seeds of oilseed plants such as castor [1] and rapeseed [2]. Seed germination, a critical developmental phase, is itself influenced by environmental factors, particularly soil water potential and temperature [3]. The combined effect of these factors significantly impacts both germination success and subsequent seedling growth [4], mainly by altering the structure and composition of seed reserves, including proteins and oils [5,6].

To quantify these relationships, several models are commonly employed: the thermal time (TT) model describes the temperature–germination relationship [7,8]; the hydrotime (HT) model describes the water potential–germination relationship; and the hydrothermal time (HTT) model integrates both factors [9]. While TT modeling identifies cardinal germination temperatures, HT analysis provides physiologically and ecologically significant parameters. For instance, the base water potential (Ψb50), the value at which 50% of a seed population germinates, is a key indicator of drought tolerance, while its standard deviation (σΨb) reflects germination uniformity [10,11]. The HTT model, first conceptualized by Bradford [9,10], provides a comprehensive framework by accumulating thermal time above a base temperature (Tb) and hydro time above a base water potential (Ψb), and has proven valuable for understanding germination variation across different seed populations and substrate conditions [12].

The use of thermal time (TT), hydrotime (HT), and hydrothermal time (HTT) models enables a comprehensive analysis of germination dynamics by isolating and combining the effects of temperature and water potential on seed behavior. TT focuses on temperature dependencies, HT addresses water potential impacts, and HTT integrates these factors to reflect real-world environmental interactions.

Sesame (*Sesamum indicum* L.), a tropical and subtropical annual oilseed crop sensitive to low temperatures, is valued for its high oil content (41–63%) [13]. Its oil is rich in unsaturated fatty acids, primarily oleic and linoleic acids, and contains saturated fatty acids, such as arachidic and palmitic acids. These fatty acid profiles are not only key quality metrics for oil products but also appear to influence germination. For example, in sunflowers, genotypes with high oleic and stearic acid content required less thermal time for 50% germination, and the base temperature was inversely related to linoleic acid content [14], underscoring the critical role of fatty acid ratios in germination efficiency under specific environments. The efficacy of the HTT model in capturing such complex interactions has been demonstrated in species such as *Retam raetam*, where it accurately predicted germination timing (R^2^ = 0.90) across varying temperatures and water potentials [15].

Under optimal conditions, seed compositions, including fatty acids in oilseeds like sesame, provide the energy reserves crucial for germination and early seedling growth. This role becomes even more critical under stress. A direct comparison with the initial analysis by Balouchi et al. [16] highlights the distinct focus of this study. Their previous work primarily aimed to estimate the cardinal temperatures for germination using different regression models (beta, dent-like, and segmented). While that study established a valuable link between lipid concentrations and germination, it did not fully quantify the interactive environmental effects using process-based physiological models. This study extends that work by systematically applying and comparing thermal time (TT), hydrotime (HT), and hydrothermal time (HTT) models to quantify the interactive effects of temperature and water potential on germination. This approach provides physiologically meaningful parameters (e.g., Tb, Ψb, θHT) crucial for predicting germination under stress. Furthermore, complemented with multivariate statistical analyses, it explores the relationships between the inherent, genotypically defined lipid profiles of different cultivars and their germination model parameters. These new integrated approaches provide a more comprehensive and mechanistic understanding of the ecology of sesame germination under stress conditions.

## 2. Results

The germination data were fitted to thermal time (TT), hydrotime (HT), and hydrothermal time (HTT) models using nonlinear regression and probit analysis, as detailed in the Section 4. The resulting model parameters for each cultivar are presented below:

### 2.1. Thermal Time Modeling

**Figure 1:**
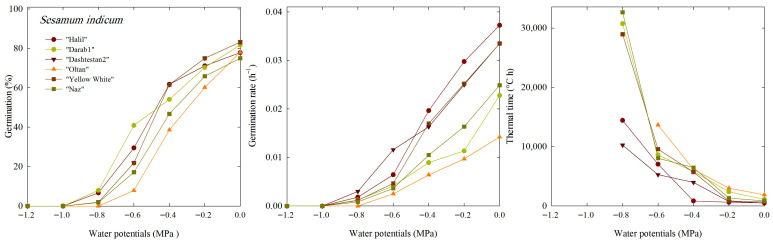
Germination percentage, germination rate, and thermal time constant under different water potentials of six cultivars of *S. indicum*.

For all cultivars, there was a noticeable decrease in germination percentage from 0 to −1.2 MPa. The germination percentage (G) was zero at water potentials of −1.0 and −1.2 MPa. Consequently, under these conditions, the germination rate (GR) was zero. At −0.8 MPa of water potential, ‘Dashtestan2’, with a value of 19.5%, showed more tolerance to a decrease in water potential (Figure 1).

The thermal time constant (θT) was calculated to quantify the effect of temperature on germination time under water potentials ranging from 0 to −0.8 MPa. θT_(50)_ reflects the number of degrees Celsius per hour required for the germination of 50% of the seed in the population. Increase in θT_(50)_ is associated with a decrease in the germination rate (GR_(50)_).

The results of the TT model parameters describing the germination response up to 50% germination (g = 50%) under different water potentials are shown for each cultivar (Table 1). The results showed that at a potential of −0.8 MPa, the ‘Naz’, ‘Darab1’, and ‘Yellow-White’ cultivars had higher θT_(50)_ values than the other cultivars, indicating a decrease in the seed germination rate of these cultivars at this water potential. There was a significant difference between different cultivars in terms of response to different water potentials and changes in θT_(50)_, so cultivars ’Darab1’ and ’Oltan’ had the highest θT_(50)_ and the lowest germination rate. In contrast, ‘Halil’ and ‘Dashtestan2’ had the highest germination rate and the lowest thermal time constant compared to other cultivars, respectively.

In ’Halil’ and ’Darab1’ cultivars, base temperature showed an increasing trend as the osmotic potential became more negative (i.e., as drought stress increased). Still, other cultivars showed different responses to base temperature and water potential. In this study, in ’Darab1’ and ’Oltan’ cultivars, the base temperature of germination was lower and the θT_(50)_ was higher than in other cultivars (Table 1).

### 2.2. Hydrotime Modeling

In fitting the hydrotime model, temperatures of 10 °C in all cultivars and 45 °C in the cultivar ‘Halil’ were excluded from the calculations due to the lack of germination, and these values are not shown in Table 2. The results of fitting the hydrotime (HT) model demonstrated that, among all cultivars, ‘Halil’ had a lower mean standard deviation of the base water potential and, as a result, the best germination uniformity, which was the highest at 20 °C (Table 2). In addition, the data show that cultivar ‘Darab1’ exhibited the lowest base water potential (Ψb) among the cultivars, indicating that it germinated at lower water potentials across most temperatures, particularly within the optimal temperature range (20–25 °C). ‘Dashtestan2’ germinates faster at 40 °C and shows about 70% germination, suggesting tolerance to drought at high temperatures during germination. Furthermore, ‘Oltan’ had the lowest germination rate and exhibited a significant response to temperature changes. For cultivar ’Naz’, the θH did not change significantly (*p* > 0.05) up to 30 °C, but it decreased sharply from 35 to 40 °C (Table 2).

### 2.3. Hydrothermal Time Modeling

The parameter values for the hydrothermal time model are shown in Table 3. The range for ‘Halil’ was limited to 15–30 °C because germination was poor outside this range, which reduced model accuracy. The cultivar ’Halil’ with θHT of 213.4 MPa·°C·h had the highest rate, while the cultivar Oltan with θHT of 2495.8 MPa·°C·h required the longest time for germination among the cultivars. The Ψb(g) values in the studied cultivars were similar, with ‘Oltan’, ‘Dashestan2’, ‘Darab1’, ‘Halil’, ‘Yellow-White’, and ‘Naz’ showing progressively lower Ψb(g). The lower this parameter is, the more drought-tolerant the seed is during germination. Cultivars with low Tb (e.g., ‘Oltan’) demonstrate better germination at low temperatures, indicating temperature could be a limiting factor in cooler conditions. Conversely, cultivars with lower Ψb (e.g., ‘Darab1’) are better suited to dry conditions, highlighting the importance of water potential in drought tolerance.

### 2.4. Multivariate Statistical Analyses

**Figure 2:**
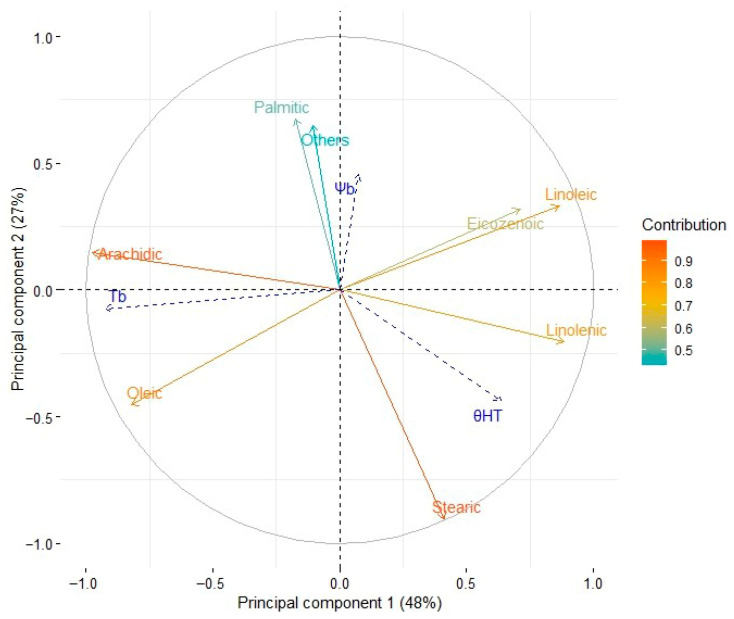
PCA on eight variables related to the mean fatty acid content of seeds and the values of three parameters (Ψb, Tb, and θHT) of a hydrothermal time model of germination for six cultivars of *S. indicum*. Normalized contribution of the variables to components 1 and 2.

**Figure 3:**
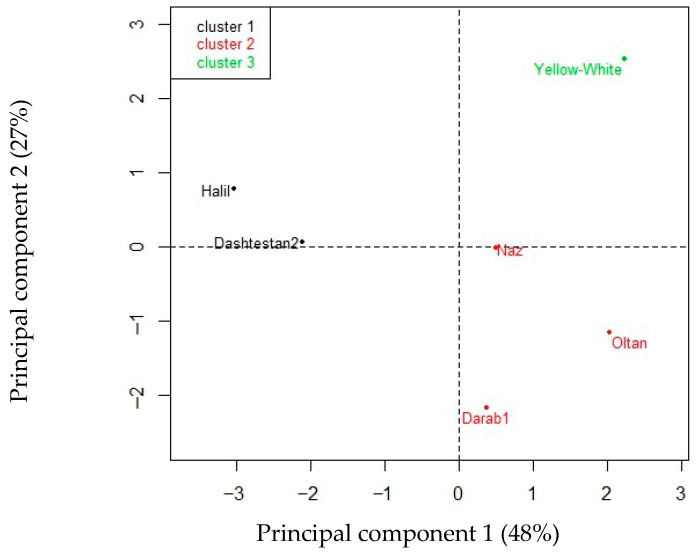
Hierarchical clustering into three groups of the six cultivars of *S. indicum* tested for seed fatty acid composition.

Principal component analysis of eight variables related to the mean fatty acid content of the seeds from the six cultivars is summarized in Figure 2 and Figure 3.

Figure 2 shows the results of a principal component analysis (PCA) performed on the germination data for the six sesame cultivars under different temperature and water potential conditions. The PCA was used to reduce the dataset’s dimensionality and highlight the underlying patterns in seed germination responses. The first principal component (PC1) accounts for the most significant variance and reflects the combined influence of temperature and water potential on germination. Positive PC1 values are associated with higher germination rates under optimal conditions, whereas negative values are associated with lower germination rates under extreme conditions. The second principal component (PC2) captures secondary variation, emphasizing cultivar-specific adaptations, such as drought tolerance or temperature sensitivity.

Five components accounted for 100% of the variability in the fatty acid profile of each cultivar. The first two components accounted for 75.6% of the variability. It was observed that five variables contributed more than 13% each to principal component 1. Two saturated fatty acids (palmitic, 20.8% and stearic, 37.6%) were the major contributors to component 2. This analysis allowed a practical condensation of the data (Figure 2).

The supplementary continuous variables Tb (base temperature), Ψb (base water potential), and θHT (hydrothermal time) showed variability in different directions within the PCA’s multidimensional space, indicating distinct contributions to the germination behavior of the cultivars. This variability was assessed by examining cos^2^ values (the squared cosine of the angle between each variable and the principal components), which measure the quality of each variable’s representation on the PCA axes.

From Figure 2, it is clear that Tb had the greatest contribution to component 1 (PC1), with a cos^2^ value of 0.855. This shows that base temperature is strongly aligned with PC1, indicating it plays a dominant role in explaining the variation captured by this axis. PC1 likely reflects variability driven by temperature-related differences in germination rates. Ψb mainly contributed to components 2 (PC2) and 3 (PC3), with cos^2^ values of 0.210 and 0.363, respectively. This indicates that base water potential is more closely linked to secondary and tertiary sources of variability, likely reflecting its role in germination under different water-stress conditions rather than direct temperature effects. θHT had moderate contributions to both PC1 and PC3, with cos^2^ values of 0.409 and 0.194, respectively. This suggests that hydrothermal time, which combines effects of temperature and water potential, influences the main variability in germination while also playing a role in more complex interactions captured by PC3. Overall, the cos^2^ values emphasize the distinct roles of each parameter in explaining the observed germination behavior. Tb strongly associates with PC1 and highlights the importance of temperature as a key factor for germination variability. In contrast, Ψb and θHT are more spread across multiple components, indicating their influence on more subtle aspects of germination under combined temperature and water potential conditions.

The relationship between θHT and fatty acids in each cultivar showed that oleic, palmitic, and arachidic acids tend to decrease. In fact, as the levels of these fatty acids increased, the hydrothermal time constant decreased; however, higher levels of linolenic and stearic acids led to an increase in θHT. These findings confirm that cultivars ’Halil’ and ’Dashtestan2’ have higher germination percentages and rates, which are related to their greater oleic acid content. It suggests that cultivars with high levels of oleic acid (around 41%) and linoleic acid (around 43%) exhibit better germination, establishment, and drought tolerance.

Hierarchical clustering of principal components (HCPC) grouped the six sesame cultivars into three clusters (Figure 3). Despite the small number of cultivars, the HCPC classified them in a way that shows similarities within clusters and differences between clusters. In fact, the two cultivars in cluster 1 had the highest Tb values (above 13 °C), which were linked to the highest percentages of arachidic acid and the lowest percentages of linolenic fatty acids in the seeds.

The three cultivars in cluster 2 had the highest values of θHT (760–2495 MPa·°C·h) for germination, while simultaneously exhibiting opposite percentages of palmitic (<9%) and stearic (>5%) acids, which contrasts with the contents of the other cultivars.

The only cultivar in cluster 3 had the lowest values of base water potential (Ψb) and oleic acid (39%) and the highest percentage of linoleic acid (44.7%).

## 3. Discussion

The initial vegetative growth and optimal establishment of plants are determined mainly by seed germination behavior under different environmental conditions. While measuring germination rates at specific temperatures can reveal some variability, this approach has limitations. Germination modeling provides detailed parameters, such as base temperature (Tb) and hydrothermal time (θHT), which are critical for predicting germination under a broader range of conditions. Without modeling, observed variability at a single temperature may not reflect broader patterns across temperature and water potential gradients. Therefore, modeling is essential for a robust, predictive understanding of germination across diverse environmental scenarios. The use of HT, TT, and HTT models can be highly effective in agricultural management programs, provided that seed germination responses are quantified correctly across diverse ecological conditions [17].

This study extends the analysis initially presented by Balouchi et al. [16], which established a foundational link between lipid concentrations and germination responses in the same six sesame cultivars. While the previous study provided valuable initial insights, its analysis was primarily focused on the main effects of temperature and water potential on final germination percentages and rates. In contrast, the present re-analysis employs a more advanced and quantitative framework by systematically applying and comparing TT, HT, and HTT models. This approach allowed us to extract fundamental physiological parameters (Tb, Ψb, θHT) that quantify the cultivars’ intrinsic thresholds and vigor. Furthermore, by complementing the modeling with multivariate statistical analyses (PCA and HCPC), we were able to directly explore and visualize the relationships between the genotypically-defined fatty acid profiles and the estimated model parameters. Consequently, this study provides a more integrated and mechanistic understanding of how specific seed biochemical traits influence germination ecology under stress, moving beyond correlation to a model-based, quantitative assessment that offers predictive power for cultivar selection.

In our study, the germination of six *S. indicum* cultivars was significantly influenced by both temperature and water potential [18]. As the temperature approached the optimal level, germination percentage increased, while it decreased at sub- and supra-optimal temperatures [19]. This pattern aligns with known physiological responses: low temperatures cause enzyme inactivation, and high temperatures lead to protein degradation [20]. Genotypic variation in response to temperature was evident, similar to findings in rice (*Oryza sativa* L.), where genotype-temperature interactions significantly affected germination traits [21]. Specifically, cultivars ‘Darab1’ and ‘Oltan’ showed lower base temperatures but higher θHT values than other cultivars (Table 2), indicating greater resistance to low-temperature stress. This is consistent with previous research on sesame that demonstrated delayed germination at low temperatures [22].

The thermal time (θT) for the six sesame cultivars varied greatly (498.4 to 32,638.6 °C·h) across different osmotic potentials. As water potential decreased, θT increased significantly, while germination rates dropped sharply (Table 1). The lowest θT and highest germination rates were observed at 0 MPa, whereas the highest θT and lowest germination rates occurred at −0.8 MPa. The lower θT in ‘Halil’ under stress conditions indicates a faster germination rate, making it especially suitable for arid regions. These findings confirm previous results in *Melissa officinalis*, where a decrease in water potential from −0.4 to −0.8 MPa led to a sharp rise in θT [23]. The variation in θT responses among sesame cultivars underscores their different sensitivities to drought stress during germination.

The hydrotime model uncovered key aspects of drought tolerance mechanisms. Natural variation in base water potential (Ψb) among individual seeds within a population directly influences germination rate and uniformity [10]. When the ambient water potential exceeds the average Ψb, germination is slower and less uniform, whereas larger differences lead to faster, more synchronized germination. In our study, drought stress caused by polyethylene glycol reduced water absorption and endosperm hydrolysis, lowering germination percentage [24]. Cultivars ‘Halil’ and ‘Darab1’ showed a link between increasing base temperature and decreasing water potential, although other cultivars responded differently (Table 1). ‘Darab1’s ability to germinate under lower water potentials, especially at optimal temperatures, demonstrates its superior drought tolerance among the cultivars studied.

In our research, the cultivar ’Halil’ showed the highest germination uniformity and the lowest average standard deviation of the base water potential, which was notably evident at the optimal temperature of 20 °C (Table 2). Cultivar ’Halil’ outperformed others at ideal water potentials, displaying greater uniformity and resilience. Additionally, the lowest base water potential for germination indicates that the cultivar has the highest drought tolerance, while the highest drought tolerance was attributed to the cultivar ’Darab1’ (Table 2). Similar to the results of this study, Soltani and Farzaneh [25] examined the HT model of cotton seeds and found that θH varied with cotton (*Gossypium hirsutum* L.) seed mass, ranging from 14.7 to 105.5 MPa h.

As temperature decreases, especially at suboptimal levels, the θH of sesame seeds increases, while the germination rate drops. At optimal temperatures, water potentials above the base water potential lead to higher enzyme activity, faster water absorption, and quicker seedling emergence [26]. The range of hydrotime constants (θH) and base water potentials (Ψb) observed among sesame cultivars is similar to interspecific variations reported in other studies. For instance, Farahinia et al. [27] reported θH values ranging from 6.8 to 24.3 MPa·h across *Trachyspermum ammi* ecotypes. At the same time, our study shows a broader range, highlighting the considerable intraspecific variability in sesame. The base water potential for germination of 50% of the seed cultivar ranged from −0.12 to −0.32 MPa, and the standard deviation in the normal distribution of germination ranged from 0.13 to 0.31.

The HTT models predict germination timing by increasing germination percentages in subpopulations during the seeding period, based on temperature, across intermittent or specific moisture periods. These moisture periods are defined by the substrate’s water content, which the seed is expected to imbibe and germinate [28]. Incorporating the hydrothermal time model revealed meaningful relationships between fatty acid composition and germination behavior. Oleic, palmitic, and arachidic acids showed negative relationships with θHT but positive correlations with germination rate. Conversely, linolenic, linoleic, and stearic acids displayed positive relationships with θHT and negative correlations with germination rate (Figure 3). These patterns can be explained by the oxidative stability of different fatty acid cultivars, with higher oleic and palmitic acid contents likely benefiting from their greater stability and resistance to oxidation, supporting more efficient germination. In contrast, higher linoleic and linolenic acid levels may reduce seed reserve utilization efficiency by accelerating oxidation, thereby slowing germination [29,30]. These findings support earlier studies in sunflowers (*Helianthus annuus* L.), showing that seed oil composition influences thermal-time requirements [14], indicating a conserved physiological mechanism across oilseed species. Similar to this research, González Belo et al. [14] reported that breeding sunflower plants for higher oleic acid and lower linoleic acid in the oil affects germination performance at low temperatures.

Correlation analysis provided statistical support for these relationships, with oleic acid showing a strong positive correlation with germination rates (r = 0.82, *p* < 0.01), while linoleic and linolenic acids showed significant negative correlations (r = −0.78 and r = −0.70, respectively; *p* < 0.01). Saturated fatty acids, such as arachidic acid, showed weak correlations (r = −0.25, *p* = 0.18), suggesting minimal direct influence on germination rates. The θHT varied significantly among cultivars (213.4–2495.8 MPa·°C·h), reflecting substantial differences in germination rates (Table 2). The increase in Ψb50 at temperatures outside the optimal range may serve as an adaptive strategy that reduces hydrotime requirements and improves seed survival under adverse conditions [31].

Our findings highlight the importance of considering the inherent genetic and biochemical makeup of seeds when selecting crops. The correlations we found between fixed fatty acid profiles and germination parameters suggest that a cultivar’s baseline seed composition can serve as a key predictor of its germination performance under stress. Even in arid regions where the fatty acid profile for a particular cultivar remains stable, selecting a cultivar with a specific innate profile (such as high oleic acid for improved germination rates) can be an effective strategy to boost establishment under local stress conditions. Identifying cultivars such as ‘Darab1’ for drought tolerance and ‘Dashtestan2’ for high-temperature resilience provides practical insights for improving sesame cultivation amid climate variability.

The multiplicative form of the HTT model proved useful for measuring the combined effects of temperature and water potential on seed germination. This method, successfully used in previous studies [9,23], offered reasonable estimates of the complex biological processes involved. However, we recognize that multiplicative models may not always be ideal, especially when temperature-water potential interactions are not independent or linear. Investigating alternative models could provide deeper insights into the biological mechanisms of seed germination [7,8]. Despite these limitations, multiplicative models remain valuable tools for both practical and theoretical applications in seed biology. The rise in θT with decreasing water potential seen in our study matches findings in other species. For example, Nozarpour et al. [23] observed a sharp increase in the thermal time constant for *Melissa officinalis* seeds as water potential dropped from −0.4 to −0.8 MPa, a pattern consistent with our results in sesame.

Our findings underscore the importance of considering both genetic and biochemical factors in crop selection. The correlations between inherent fatty acid profiles and germination parameters indicate that a cultivar’s baseline seed composition can predict its germination performance under stress. Therefore, even in arid regions where fatty acid profiles remain stable, selecting cultivars with specific innate profiles (e.g., high oleic acid for improved germination rates) is a viable strategy to enhance establishment under local stress conditions. By identifying ‘Darab1’ for drought tolerance and ‘Dashtestan2’ for high-temperature resilience, this study provides actionable insights for improving sesame cultivation amid climate variability.

While our study focused on the germination phase and provided critical baseline parameters (Tb, θT), we recognize that the total temperature requirement for full generative maturity represents a different phenological stage. The parameters we established are fundamental for predicting initial seedling emergence, which is a prerequisite for accumulating additional heat units toward maturation. Future research integrating these germination models with subsequent developmental-stage models could yield a comprehensive thermal-time framework for the entire sesame life cycle, thereby enhancing our ability to predict crop performance under changing environmental conditions.

## 4. Materials and Methods

### 4.1. Plant Material

Seeds of six cultivars of sesame (‘Halil’, ‘Darab1’, ‘Dashtestan2’, ‘Oltan’, ‘Yellow-White’, and ‘Naz’) were provided by “The Seed and Plant Improvement Institute” in Karaj, Iran. The seeds were from the previous growing season and had been stored in cold storage post-harvest to maintain viability until the start of the experiments. The characteristics of the seed lots, including the specific production year, total oil content, and fatty acid profiles, were described previously [16] (shown in Supplementary Tables S1–S3).

### 4.2. Germination Responses

The protocol was previously detailed in Balouchi et al. [16]. Briefly, the experiment was performed using a completely randomized design at the Seed Technology Laboratory, Faculty of Agriculture, Yasouj University, Iran, with four replications. The first factor was sesame cultivars, and the second factor included seven levels of water potential (0, −0.2, −0.4, −0.6, −0.8, −1.0, and −1.2 MPa), tested across eight temperature levels (10, 15, 20, 25, 30, 35, 40, and 45 °C) under 12/12 h light/dark conditions.

The different water potential levels were obtained by using solutions of polyethylene glycol 6000 (Merck, Darmstadt, Germany) dissolved in distilled water, as described by Michel and Kaufmann [32]. They are presented in Table 4.

The seeds were sterilized in a 1% sodium hypochlorite solution for 60 s before being transferred to glass Petri dishes (9 cm diameter). For each water potential level, 10 mL of osmotic solution was added to each Petri dish, and 10 mL of distilled water was used for the 0 MPa treatment. To minimize evaporation and maintain a constant water potential, all Petri dishes were sealed with Parafilm. Germinated seeds were counted every eight hours, and a seed was considered germinated when the radicle protruded at least 2 mm. After seven days, the seed germination percentage (G) and rate (GR) were calculated [33].

To quantify the germination response to temperature, the TT model (Equation (1)) was used [9]:

(1) $$\theta T\left(g\right)=\left(T-Tb\right)\cdot t(g)\;GR(g)=\frac{1}{t(g)}=\frac{\left(T-Tb\right)}{\theta T(g)}$$

where θT, is the thermal time constant (°C·h); T, is temperature (°C); Tb, is base temperature (°C); t(g), in hours, is the time needed to reach a particular fraction of germinated seed in the lot (G, %); GR, is the germination rate (h^−1^) at t(g) [34].

To quantify the response of germination rate to water potential, the HT model (Equation (2)) was used [10]:

(2) $$\theta H=(\Psi -\Psi b(g))\cdot t(g)\;GR(g)=\frac{1}{t(g)}=\frac{\left(\Psi -\Psi b(g)\right)}{\theta H}$$

where θH is hydrotime constant (MPa h); Ψ is actual seed water potential (MPa); Ψb(g) is base water potential (MPa).

To quantify the response of germination rate to temperature and water potential, the HTT model (Equation (3)) was used [9]:

(3) $$\theta HT=\left(\Psi -\Psi b\left(g\right)\right)\cdot (T-Tb)\cdot t(g)\;GR(g)=\frac{1}{t(g)}=\frac{\left(\Psi -\Psi b\left(g\right)\right)\cdot (T-Tb)}{\theta HT}$$

where θHT is the hydrothermal time constant (MPa·°C·h).

Probit analysis was used to estimate the parameters (Equation (4)):

(4) $$Probit\left(g\right)=\;\frac{\left\{\left[\frac{\Psi -\theta HT}{\left(T-Tb\right).tg}\right]{-\Psi b}_{\left(50\right)}\right\}}{\sigma_{\Psi b}\;}$$

where Ψb(50) is the value of Ψb when half of the seeds of a lot have germinated, and σ_Ψb_ represents the standard deviation in base water potential between the seeds contained in the population. In this way, all observed germination percentages at both temperatures and all Ψ levels were regressed against Ψ − θHT/(T − Tb) tg, varying the value of θHT until the best fit was obtained.

### 4.3. Statistical Analysis

The statistical analysis was conducted in two main stages. First, parameters for the thermal-time, hydro-time, and hydrothermal-time models (e.g., θT, Tb, θH, σΨb, Ψb, θHT, RMSE, and R^2^) were estimated using nonlinear regression and probit analysis, with model fitting performed in SAS (PROC NLIN, SAS 9.1, SAS Institute Inc., Cary, NC, USA) and R (R software version 4.3.2, Available online: https://www.r-project.org (accessed on 31 October 2023), packages: nlstools- R Core Team), providing the quantitative basis for germination behavior. Observations with no further increase in germination percentage during the rest of the germination test of a given treatment were, therefore, excluded from the data set before analysis. Subsequently, to explore the complex interrelationships between all estimated model parameters and the seed fatty acid profiles, multivariate statistical analyses were performed. This included principal component analysis (PCA) to reduce dimensionality and visualize the significant patterns of variation among cultivars, followed by hierarchical clustering on principal components (HCPC) [35] to group the cultivars based on their combined characteristics objectively.

PCA and hierarchical clustering on principal components (HCPC) were performed on the six cultivars, described by eight variables related to their seeds’ mean fatty acid content and three parameters of the hydrothermal time model of germination. All these analyses were performed with R (R software version 4.3.2, Available online: https://www.r-project.org (accessed on 31 October 2023), packages: base, stats, FactoMaineR, and nlstools- R Core Team) [36].

## 5. Conclusions

The use of models related to quantifying germination responses to temperature and moisture conditions can help evaluate the quality of seed cultivars and their tolerance to stress conditions, such as low temperatures and drought. Similarly, identifying seed compounds and their relationship with germination percentage and seedling establishment under different environmental conditions should be effective in achieving high yields. In this research, germination percentage and rate were investigated as two critical parameters affecting the quality of sesame seed cultivars. The results showed that environmental conditions, including temperature and various water potentials, significantly influenced these indicators. Differences in fatty acid composition profiles among cultivars or seed lots may underlie the observed variations in response. Cultivars that respond favorably to low base water potential, such as ‘Darab1’, are drought-tolerant. Cultivars with a low base temperature (below 8 °C), such as ‘Oltan’, ‘Yellow-White’, and ‘Naz’, can be used for earlier spring planting. Based on ‘Deshtestan2’’s capacity for germination at high temperatures and low water potentials, this cultivar could be valuable for cultivation in conditions where others might struggle or fail to germinate. Although this research offers insights into seed responses under different environmental conditions, further field studies are needed to confirm these findings. This study demonstrates the usefulness of TT, HT, and HTT models in predicting seed germination behavior under variable environmental conditions. It is important to note that, while actual germination timing in the field depends on dynamic climatic sequences, these models capture each cultivar’s intrinsic physiological thresholds (Tb, Ψb) and vigor (θHT). Since these traits are genetically determined and reflected in stable seed characteristics such as fatty acid profiles, the comparative resilience or sensitivity of cultivars, as identified by the models, provides a reliable basis for selection under diverse and changing climates. Additionally, deeper analysis of existing data using incremental and deterministic modeling, along with multivariate statistical analyses, offered richer insights into sesame seed germination. This study highlights the relationship between seed fatty acid composition and environmental factors, advancing our understanding of germination mechanisms and offering valuable guidance for selecting sesame cultivars suitable for varying climatic conditions.

## Figures and Tables

**Table 1:** Thermal-time parameters for describing the germination response under different water potentials (Ψ) and temperatures of six cultivars of *S. indicum*.

Cultivars	Ψ (MPa)	θT_(50)_ (°C h)	T_b_ (°C)	RMSE	R^2^	GR_(50)_ (h^−1^)
Halil	0.0	498.4	11.72	105.48	0.359	0.0372
	−0.2	615.9	13.21	45.53	0.855	0.0297
	−0.4	837.0	13.76	105.58	0.820	0.0196
	−0.6	7027.5	11.95	61.51	0.923	0.0064
	−0.8	14,413.7	14.82	169.13	0.506	0.0018
Darab1	0.0	1090.7	4.00	21.18	0.631	0.0227
	−0.2	2375.5	6.00	44.91	0.584	0.01133
	−0.4	5781.3	8.00	389.05	0.722	0.00893
	−0.6	8602.6	14.90	426.7	0.343	0.00409
	−0.8	30,735.7	20.00	0.00	0.000	0.00124
Dashtestan2	0.0	509.5	12.65	30.02	0.817	0.03344
	−0.2	751.6	13.08	65.55	0.755	0.02497
	−0.4	3963.2	11.08	62.91	0.965	0.01631
	−0.6	5270.8	11.05	56.17	0.843	0.01159
	−0.8	10,250.1	12.38	120.99	0.870	0.00299
Oltan	0.0	1868.2	5.00	36.43	0.861	0.01420
	−0.2	2978	5.00	77.50	0.721	0.00967
	−0.4	6140.3	10.00	47.01	0.978	0.00638
	−0.6	13,640.4	9.94	113.52	0.809	0.00249
	−0.8	-	-	-	-	-
Yellow-White	0.0	708.6	5.00	19.18	0.775	0.03345
	−0.2	834.4	10.00	19.10	0.855	0.02518
	−0.4	5702.6	10.00	229.61	0.581	0.01698
	−0.6	9561.7	10.00	137.77	0.941	0.00464
	−0.8	28,928.7	3.100	0.00	0.000	0.00145
Naz	0.0	843.9	10.00	22.77	0.796	0.02486
	−0.2	1325.4	10.00	20.78	0.932	0.01633
	−0.4	6449.6	5.00	45.97	0.914	0.01048
	−0.6	8039.9	8.00	90.98	0.841	0.00362
	−0.8	32,638.6	5.00	0.00	1.000	0.00083

Note: RMSE: Root mean square error, R^2^: Coefficient of determination, Ψ: Water potential, θT_(50)_ thermal time constant for 50% germinated seeds in a population, Tb: Base temperature, GR_(50)_: Germination rate for 50% germinated seeds in a population.

**Table 2:** Parameters of the hydrotime model for six cultivars of *S. indicum*.

Cultivar	Temperature (°C)	θ_H_ (MPa h)	Ψ_b50_ (MPa)	σ_Ψb50_ (MPa)	Predicted G (%)	Observed G (%)	R^2^	RMSE
Halil	15	46.984	−0.309	0.156	37	41	0.99	10.18
	20	28.991	−0.533	0.154	35	36	0.90	9.99
	25	17.072	−0.794	0.190	38	33	0.84	13.98
	30	11.553	−0.665	0.225	49	48	0.90	11.43
	35	5.604	−0.621	0.165	48	50	0.85	13.65
	40	4.161	−0.536	0.242	47	47	0.84	13.40
	45	-	-	-	-	-	-	-
Darab1	15	31.8	−0.283	0.099	29	31	0.94	6.26
	20	79.98	−1.073	0.312	49	49	0.83	13.02
	25	32.55	−1.016	0.330	54	53	0.89	11.68
	30	30.40	−0.753	0.255	49	51	0.83	13.84
	35	18.51	−0.650	0.219	51	49	0.79	15.13
	40	10.89	−0.548	0.273	63	61	0.68	15.68
	45	16.64	−0.144	0.159	23	25	0.98	3.61
Dashtestan2	15	45.71	−0.314	0.289	33	40	0.63	19.98
	20	50.61	−0.922	0.257	48	47	0.87	11.98
	25	31.55	−1.177	0.340	48	47	0.87	13.47
	30	17.99	−0.752	0.223	38	39	0.95	7.66
	35	8.155	−0.827	0.362	66	63	0.88	10.28
	40	4.973	−0.573	0.222	72	72	0.55	18.22
	45	12.33	−0.123	0.144	22	24	0.97	4.98
Oltan	15	65.61	−0.428	0.132	26	30	0.98	4.73
	20	36.17	−0.518	0.247	32	35	0.87	12.60
	25	25.72	−0.733	0.274	49	49	0.85	13.12
	30	81.86	−1.693	0.473	67	60	0.86	15.64
	35	7.477	−0.153	0.097	55	56	0.89	11.32
	40	69.60	−1.331	0.560	59	58	0.94	7.38
	45	23.16	−0.018	0.491	20	20	0.97	1.99
Yellow-White	15	26.83	−0.370	0.228	33	33	0.82	13.93
	20	29.29	−0.576	0.211	42	40	0.78	14.99
	25	17.49	−0.705	0.236	36	38	0.89	12.16
	30	18.33	−0.613	0.195	46	49	0.87	13.22
	35	4.568	−0.557	0.271	61	63	0.77	17.49
	40	10.08	−0.942	0.379	51	64	0.86	19.34
	45	11.11	−0.050	0.156	24	27	0.91	6.04
Naz	15	19.01	−0.196	0.149	25	24	0.90	10.7
	20	36.68	−0.656	0.222	50	48	0.81	14.94
	25	21.24	−0.687	0.183	52	52	0.88	13.28
	30	23.39	−0.827	0.282	47	45	0.91	9.87
	35	3.396	−0.344	0.171	51	51	0.89	12.18
	40	4.061	−0.333	0.3420.	47	52	0.67	19.41
	45	11.11	0.079	0.144	20	20	0.97	3.65

Note: θ_H_: Hydrotime constant, Ψ_b50_: Base water potential for 50% germinated seeds in a population, σ_Ψb50_: Standard deviation of water potential for 50% germinated seeds in a population, R^2^: Coefficient of determination; RMSE: root mean square error; GP pre: Predict germination percentage; GP obs: Observed germination percentage.

**Table 3:** Parameters of the hydrothermal time (HTT) model for six cultivars of *S. indicum*.

Cultivars	Temperature (°C)	T_b_ (°C)	Ψ_b_ (MPa)	R^2^	RMSE	θHT (MPa·°C·h)	SEM
Halil	15–30	13.096	−0.575	0.92	0.0054	213.4	13.40
Darab1	15–45	10.589	−0.638	0.69	0.0068	760.2	77.90
Dashtestan2	15–45	13.576	−0.670	0.53	0.0155	356.9	48.92
Oltan	15–45	7.485	−0.691	0.31	0.0059	2495.8	529
Yellow-White	15–45	7.638	−0.545	0.55	0.0138	561.4	95.11
Naz	15–45	7.60	−0.446	0.36	0.0073	1049	369.1

Note: T_b_: Base temperature, Ψ_b_: Base water potential, θHT: Hydrothermal time constant, R^2^: Coefficient of determination; RMSE: Root mean square error; SEM: Standard error of the mean.

**Table 4:** Concentration of polyethylene glycol-6000 in distilled water (g/L) for the germination response to temperature at different water potentials of six cultivars of *S. indicum*.

Temperature (°C)	Water Potential (MPa)					
	−0.2	−0.4	−0.6	−0.8	−1.0	−1.2
	Concentration of polyethylene glycol-6000 in distilled water (*g*:*L*)					
10	99.8	153.9	196.0	231.8	263.3	291.9
15	105.7	161.4	204.5	241.0	273.3	302.5
20	112.3	169.5	213.7	251.1	284.1	313.9
25	119.6	178.4	223.7	262.0	295.8	326.3
30	127.8	188.3	234.7	274.0	308.5	339.7
35	137.2	199.1	246.7	286.9	322.4	354.3
40	147.5	211.2	260.1	301.1	337.6	370.5
45	159.3	224.8	275.0	317.1	354.4	388.2

## Data Availability

The data presented in this study are available on request from the corresponding author (H.B.). The data are not publicly available due to privacy and ethical restrictions.

## Supplementary Materials

The following supporting information can be downloaded at: https://www.mdpi.com/article/10.3390/plants14223422/s1, Table S1. Characteristics of sesame varieties studied. Table S2. Essential characteristics of sesame seeds were tested. Table S3. Fatty acid profiles (%) of sesame seeds tested.

## Abbreviations

The following abbreviations are used in this manuscript:

HTT	Hydrothermal time
TT	Thermal time
HT	Hydrotime
GR	Germination rate
θH	Hydrotime constant
θHT	Hydrothermal time constant
G	Germination percentage
θT	Thermal time constant
