# Supplementary material for: Deciphering the Germination Behavior of Sesame Cultivars: The Interplay of Hydrothermal Time Model Parameters and Seed Fatty Acid Profiles
Source: Plants (Basel). 2025 Nov 8;14(22):3422. doi: 10.3390/plants14223422 (PMC12656520; doi:10.3390/plants14223422)
Supplement: Supplementary file 1 [file plants-14-03422-s001.zip › plants-3928651-supplementary.pdf]

plants

MDPI

Supplementary Tables of the Article

Deciphering the Germination Behavior of Sesame Cultivars: The Interplay of Hydrothermal Time Model Parameters and Seed Fatty Acid Profiles

Citation: Balouchi, H.; Soltani Khankahdani, V.; Piri, R.; Moradi, A.; Gholamhoseini, M.; Heydari, S.Z.; Ahmed, L.Q.; Escobar-Gutiérrez, A.J.; Dedicova, B. Deciphering the Germination Behavior of Sesame Cultivars: The Interplay of Hydrothermal Time Model Parameters and Seed Fatty Acid Profiles. *Plants* **2025**, *14*, x. https://doi.org/10.3390/xxxxx

Supplementary Table S1. Characteristics of sesame varieties studied.

Cultivars	Halil	Darab1	Dashtestan2	Oltan	Yellow-White	Naz
Year of introduction	2013	2009	2006	1999	2006	2001
Branching	Branched	Branched	Branched	Branched	Branched	Single branch
Seed color	Brown	light brown	light brown	dark brown	Light cream	Cream
Production Year	2017–2018	2017–2018	2017–2018	2017–2018	2017–2018	2017–2018

Supplementary Table S2. Essential characteristics of sesame seeds were tested.

Cultivars	Germination Percentages	Thousand Seed Weight (g)	Seed Moisture Content (%)	Oil Percentages
Halil	99	3.8	6.07	58.25
Darab1	98	3.12	5.15	54.77
Dashtestan2	99	3.44	5.37	65.05
Oltan	98	3.25	4.87	57.54
Yellow-white	99	2.91	5.85	52.66
Naz	99	2.85	5.16	54.09

Supplementary Table S3. Fatty acid profiles (%) of sesame seeds tested.

Cultivars	Palmitic	Arachidic	Stearic	Oleic	Linoleic	Linolenic	Eicozenoic	Others
Halil	9.1	0.6	4.3	43.3	40.3	0.2	0.1	2.1
Darab1	8.9	0.4	5.7	43.5	40.9	0.3	0.1	0.0
Dashtestan2	9.5	0.6	4.8	45.3	39.3	0.3	0.1	0.1
Oltan	9.0	0.4	5.8	40.7	43.8	0.3	0.1	0.0
Yellow-white	9.5	0.4	4.3	38.9	44.7	0.3	0.1	1.8
Naz	8.9	0.4	5.0	41.9	40.9	0.3	0.1	2.4
